# *Lactobacillus acidophilus* impairs the establishment of pathogens in a subgingival multispecies biofilm

**DOI:** 10.3389/fdmed.2023.1212773

**Published:** 2023-07-31

**Authors:** Manuela Rocha Bueno, Gustavo Dudu-Silva, Tatiane Tiemi Macedo, Aline Paim de Abreu Paulo Gomes, Arthur Rodrigues Oliveira Braga, Lucas Daylor Aguiar Silva, Bruno Bueno-Silva

**Affiliations:** ^1^ Department of Microbiology, Institute of Biomedical Sciences, University of São Paulo, São Paulo, Brazil; ^2^ Dental Research Division, Guarulhos University, Guarulhos, Brazil; ^3^ Department of Bioscience, Piracicaba Dental School, University of Campinas (UNICAMP), Piracicaba, Brazil

**Keywords:** subgingival biofilm, periodontitis, *Lactobacillus acidophilus*, bacteria, probiotics

## Abstract

The present study evaluated the antibiofilm effects of *Lactobacillus acidophilus* within a subgingival multispecies biofilm. *Lactobacillus acidophilus* (La5) at 1 × 10^2^, 1 × 10^4^, and 1 × 10^6^ were included at the beginning of biofilm formation, which lasted 7 days. The biofilms comprised 33 periodontitis-related bacterial species and the Calgary Biofilm device was used. At the end, DNA–DNA hybridization (checkerboard) was performed. A Kruskal–Wallis test followed by a Dunn *post hoc* test were performed (*p* ≤ 0.05). La5 at 1 × 10^4^ and 1 × 10^6^ reduced the total counts of biofilm and the proportions of red and green complexes when compared to the control biofilm without La5 (*p* ≤ 0.05). La5 at 1 × 10^4^ increased the proportions of *Actinomyces* complex compared to the controls (*p* ≤ 0.05). Both La5 at 1 × 10^4^ and 1 × 10^6^ decreased levels of 20 and 14 distinct species, respectively, including *Porphyromonas gingivalis*, *Prevotella intermedia*, *Fusobacterium nucleatum polymorphum*, and *Parvimonas micra* compared to the control (*p* ≤ 0.05). Only La5 at 1 × 10^4^ reduced the levels of *Tannerella forsythia*, *Fusobacterium periodonticum*, and *Aggregatibacter actinomycetencomytans* compared to the control (*p* ≤ 0.05). *L. acidophilus* inhibited establishing periodontic pathogens from red complex such as *P. gingivalis* and *T. forsythia* in a subgingival multispecies biofilm.

## 1. Introduction

Periodontitis is clinically characterized by the loss of protective and supporting tissues of the teeth. Such destruction involving loss of periodontal ligament, cement, and alveolar bone results in a proper niche to a dysbiotic microbiome, which results in an intense immune-inflammatory response (1). The dysbiosis starts without clinical signs (2) and this bacteria-inflammation binomial remains in a positive feedback loop if the patient is not properly treated. New findings regarding periodontal disease have changed the perspective regarding its etiology and the role of those considered “periodontal pathogens,” showing a more diverse and complex periodontitis-associated microbiota, related to dysbiosis, i.e., a shift in the proportion of beneficial and pathogenic microorganisms that disrupts the homeostasis seen in health (3).

Traditionally, periodontal treatment requires control of risk factors (such as diabetes, smoking, insufficient biofilm control), mechanical debridement of affected surfaces, and administration of systemic antibiotics in severe cases (4); however, studies show that due to a biofilm characteristic called resilience, after 1 year of treatment, pathogenic bacteria tend to increase in proportion, and this may lead to disease recurrence (5). In this regard, several adjunctive therapies have been studied, such as antimicrobial photodynamic therapy (6), combinations of antibiotics (6), statins (7), and probiotics (8), in order to prevent recolonization and propagation of bacterial pathogens and/or modulate the immune response, regaining the microbiome ecological balance (9).

Probiotics are living microorganisms that may promote benefits in health (10) and they have been studied as adjunctive therapy in periodontal treatment due to their ability to decrease the colonization of pathogens and to modulate host immune response. *In vitro* studies (11, 12) have shown that gingival epithelial cells (GECs) infected either with *Aggregatibacter actinomycetemcomitans* or *Porphyromonas gingivalis* and treated with different strains of probiotics could reduce the adhesion of pathogens to GECs as well as attenuating the release of important inflammatory cytokines, such as IL-1β, CXCL-8, and GM-CSF. In addition, the postbiotics derived from lactobacilli have been shown to reduce *A. actinomycetemcomitans* biofilm formation and to decrease the expression of virulence factors, such as cytolethal distending toxin and leukotoxin (13). Moreover, an *in vivo* study using a microbial consortium to induce experimental periodontitis containing *P. gingivalis*, *Fusobacterium nucleatum*, *Prevotella intermedia*, and *Streptococcus gordonii* was successfully treated when the animals were inoculated with probiotics, by reducing alveolar bone loss (14). However, all the aforementioned studies showed that the effectiveness of treatment with probiotics depends on the strain used, since some strains have an inflammatory potential.

To add to the knowledge of the use of probiotics in the control of dysbiosis seen in periodontal disease, we evaluated whether *L. acidophillus* La5 was able to interfere in a subgingival biofilm composition through an *in vitro* model.

## 2. Materials and methods

### 2.1. Formation of multispecies subgingival biofilm

An *in vitro* multispecies biofilm was developed, as explained by Miranda et al. (15) and Pingueiro et al. (16), with inoculum alterations. The bacterial species used in the multispecies biofilm model are listed in Table 1. All bacterial species were procured from the ATCC company.

**Table 1:** Species cultivated in multispecies biofilms grouped into the bacterial complexes (17).

Multispecies biofilm strains
Actinomyces complex
*Actinomyces naeslundii* ATCC 12104
*Actinomyces oris* ATCC 43146
*Actinomyces gerencseriae* ATCC 23840
*Actinomyces israelii* ATCC 12102
Purple complex
*Veillonella parvula* ATCC 10790
*Actinomyces odontolyticus* ATCC 17929
Yellow complex
*Streptococcus sanguinis* ATCC 10556
*Streptococcus oralis* ATCC 35037
*Streptococcus intermedius* ATCC 27335
*Streptococcus gordonii* ATCC 10558
*Streptococcus mitis* ATCC 49456
Green complex
*Aggregatibacter actinomycetemcomitans* ATCC 29523
*Capnocytophaga ochracea* ATCC 33596
*Capnocytophaga gingivalis* ATCC 33624
*Eikenella corrodens* ATCC 23834
*Capnocytophaga sputigena* ATCC 33612
Orange complex
*Campylobacter showae* ATCC 51146
*Eubacterium nodatum* ATCC 33099
*Fusobacterium nucleatum vincentii* ATCC 49256
*Parvimonas micra* ATCC 33270
*Fusobacterium nucleatum polymorphum* ATCC 10953
*Fusobacterium periodonticum* ATCC 33693
*Prevotella intermedia* ATCC 25611
*Streptococcus constellatus* ATCC 27823
Red complex
*Porphyromonas gingivalis* ATCC 33277
*Tannerella forsythia* ATCC 43037
Other
*Streptococcus anginosus* ATCC 33397
*Streptococcus mutans* ATCC 25175
*Selenomonas noxia* ATCC 43541
*Propionibacterium acnes* ATCC 11827
*Gemella morbillorum* ATCC 27824

Tryptone soy agar plus 5% sheep blood (Probac, São Paulo, Brazil) was the medium to grow the majority of the species under anaerobic conditions (85% nitrogen, 10% carbon dioxide, and 5% hydrogen), while *Eubacterium nodatum* was cultivated on fastidious anaerobic agar plus 5% sheep blood. *Porphyromonas gingivalis* was grown on tryptone soy agar plus yeast extract and supplemented with 1% hemin, 5% menadione, and 5% sheep blood. *Tannerella forsythia* was cultivated on tryptone soy agar plus yeast extract, supplemented with 1% hemin, 5% menadione, 5% sheep blood, and 1% *N*-acetylmuramic acid. All species were grown on agar plates for 24 h and were then moved to glass tubes with BHI culture medium (Becton Dickinson, Sparks, MD, USA) enriched with 1% hemin. After 24 h of growth on conical tubes, the optical density (OD) was adjusted for the inoculum to have about 10^8^ cells/mL of each bacterial species. A dilution of individual bacterial cell suspensions was performed, and 100-µL aliquots containing 10^6^ cells from each species were mixed with 11,700 µL of BHI broth supplemented with 1% hemin and 5% sheep blood to acquire a 15-mL inoculum.

The multispecies biofilm model was established using a Calgary biofilm device in a 96-well plate (Nunc; Thermo Scientific, Roskilde, Denmark). A 150-µL aliquot of the inoculum was placed into each well, corresponding to ∼1 × 10^4^ cells of each bacterial species, except for *P. gingivalis* and *Prevotella intermedia*, whose inocula were modified to 2 × 10^4^ cells. A lid comprising polystyrene pins was utilized to cover the 96-well plate (Nunc TSP System; Thermo Scientific, Roskilde, Denmark). Coated plates were incubated at 37°C under anaerobic conditions. On day 3, the medium was replaced with fresh BHI broth supplemented with 1% hemin and 5% sheep blood, and the biofilm was maintained at 37°C under anaerobic conditions for another 4 days to achieve 7-day-old biofilms (15, 16). Three distinct experiments were performed in triplicate.

### 2.2. Preparation of *Lactobacillus acidophilus* (La5)

*L. acidophilus* La5™ (CHR Hansen Holding A/S, Hørsholm, Denmark) was used. Before the experiments, the strain was stored in 20% glycerol at −80°C. *L. acidophilus* La5 was cultivated under microaerophilic conditions in Lactobacilli MRS broth and agar (Lactobacilli MRS, Difco). Then, bacteria were grown in liquid media until the midlog phase. After that, the suspension was adjusted to an OD 590 nm ∼ 0.9, corresponding to 2 × 10^8^ CFU/mL. Then, the inoculum values of La5 were adjusted to final values of 1 × 10^2^, 1 × 10^4^, and 1 × 10^6^ CFU/mL for each group of analysis.

### 2.3. DNA–DNA hybridization (checkerboard)

Three 7-day biofilm coated pins from each group and from each experiment were washed in phosphate-buffered solution and transferred to microcentrifuge tubes containing 150 μL of TE buffer (10 mM Tris-HCl, 1 mM EDTA (pH 7.6)), followed by the addition of 100 μL of 0.5M NaOH. The tubes containing the pins and the final solution were boiled for 10 min, and the solution was neutralized with 0.8 mL of 5M ammonium acetate. The samples were analyzed individually for the presence and quantity of 33 bacterial species using the DNA–DNA hybridization technique. Briefly, biofilm samples were lysed by boiling them and by the ammonium acetate as described above. The corresponding DNA was plated onto a nylon membrane using a Minislot device (Immunetics, Cambridge, MA, USA). After attachment to the membrane, the DNA samples were placed in a Miniblotter 45 (Immunetics). Digoxigenin labeled with DNA probes of the entire genome of the subgingival species was hybridized to the individual lanes of the Miniblotter 45. The membranes were washed, and DNA probes were detected using a specific antibody against digoxigenin conjugated to phosphatase alkaline. The signals were detected using AttoPhos substrate (Amersham Life Sciences, Arlington Heights, IL, USA), and the results were obtained using Typhoon Trio Plus (Molecular Dynamics, Sunnyvale, CA, USA). Two lanes in each run contained standards with 10^5^ and 10^6^ cells of each strain. Signals obtained with the Typhoon Trio were converted into absolute counts by comparison with the standards on the same membrane. Failure to detect a signal was recorded as zero. The values obtained upon treatment with La5 were compared to those of the negative and positive controls (15, 18). The data were analyzed using a Kruskal–Wallis test followed by a Dunn *post hoc* test (*p* ≤ 0.05).

## 3. Results

**Figure 1:**
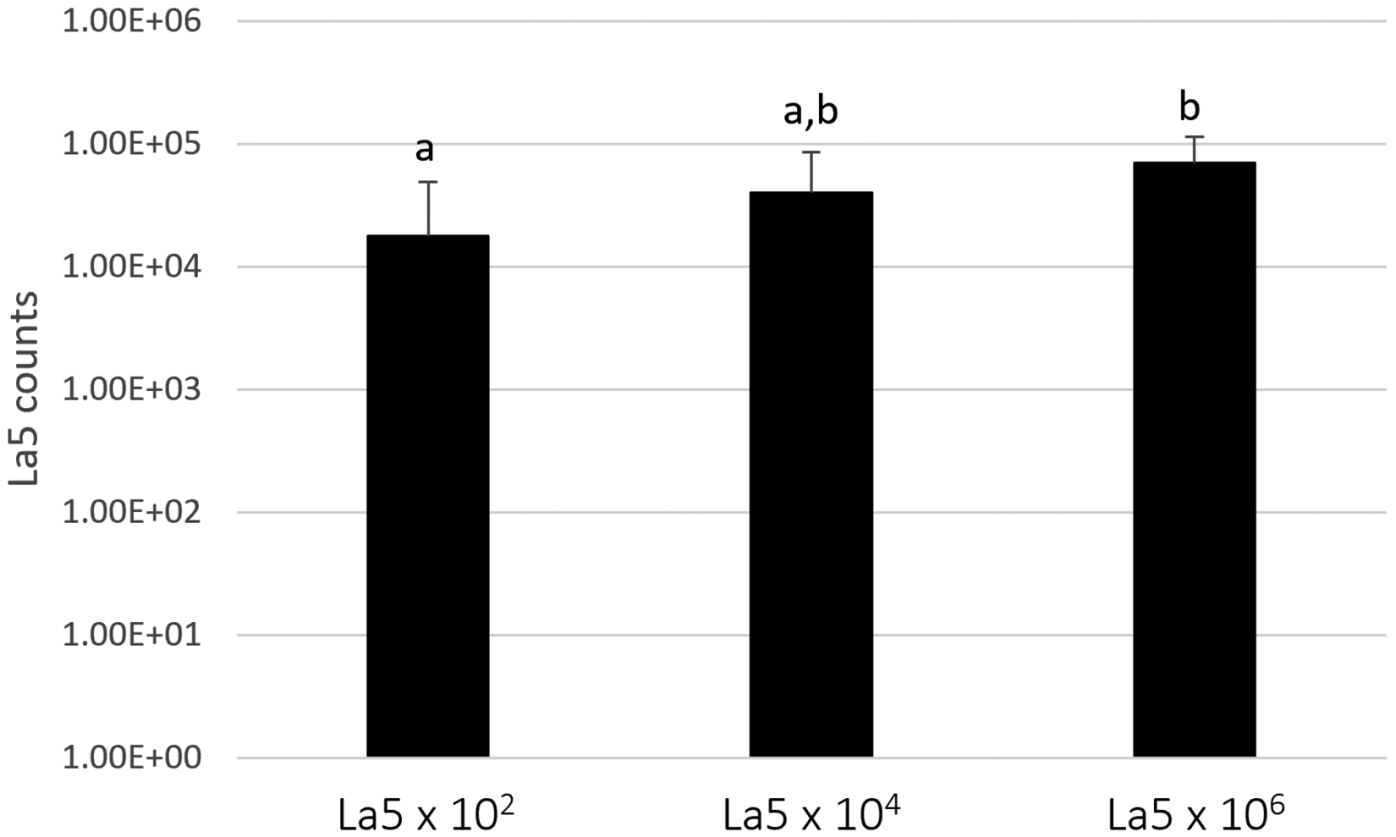
Mean counts of La5 after 7 days of biofilm formation within a multispecies biofilm. Different letters indicate statistical significance between groups by Kruskal–Wallis followed by Dunn's *post hoc* test (*p* ≤ 0.05). La5 × 10^2^ means initial inoculum with La5 × 10^2^ CFU/mL; La5 × 10^4^ means initial inoculum with La5 × 10^4^ CFU/mL; and La5 × 10^6^ means initial inoculum with La5 × 10^6^ CFU/mL.

Figure 1 shows the counts of *L. acidophilus* (La5) within the subgingival multispecies biofilm. La5 × 10^6^ presents three times more counts than La5 × 10^2^ (*p* ≤ 0.05). La5 × 10^4^ counts did not differ from any other group (*p* ≥ 0.05).

**Figure 2:**
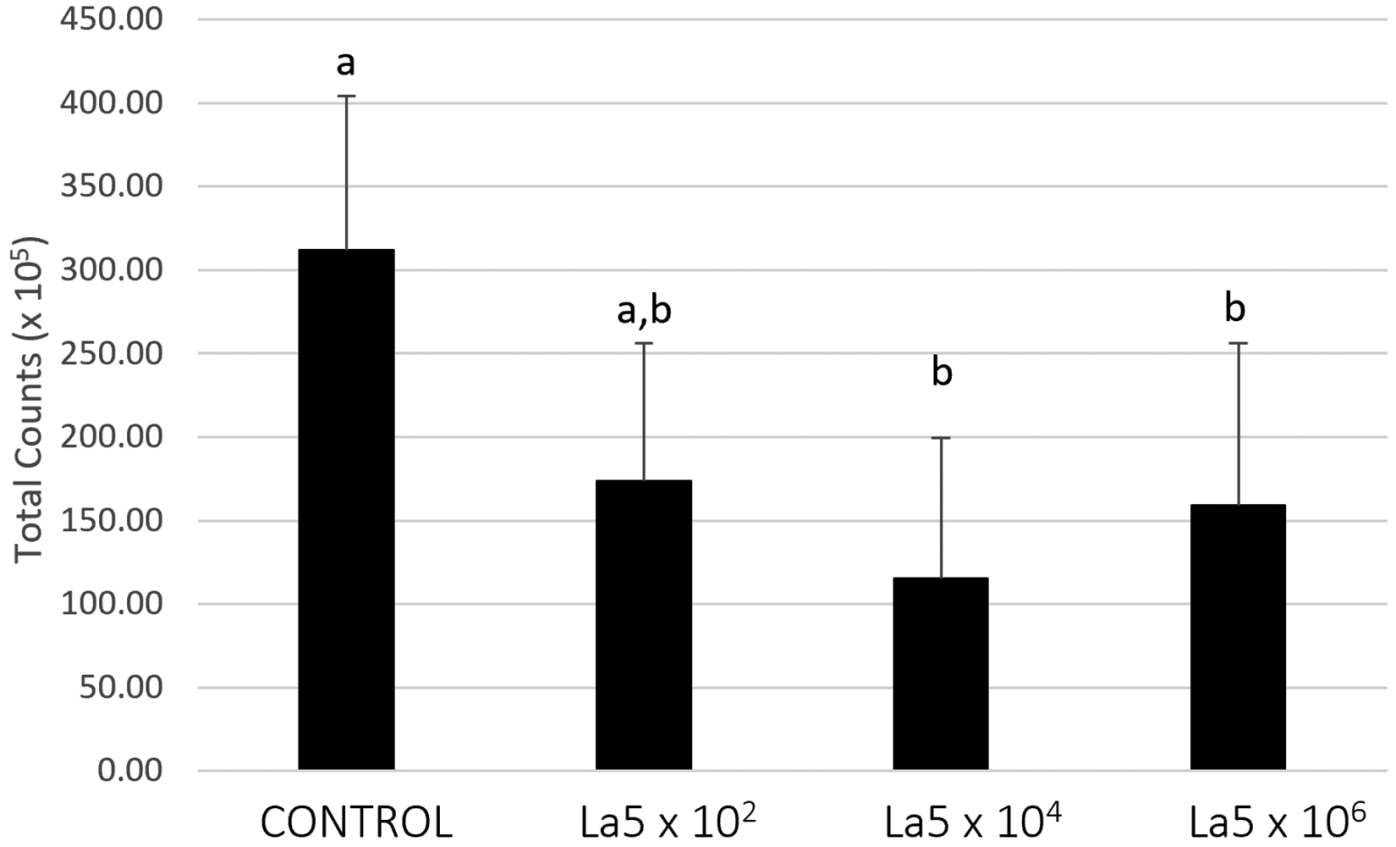
Mean total counts of multispecies biofilm without any treatment (control) and treated with La5 × 10^2^, La5 × 10^4^, and La5 × 10^6^. Different letters indicate statistical significance between groups by Kruskal–Wallis followed by Dunn's *post hoc* test (*p* ≤ 0.05). La5 × 10^2^ means initial inoculum with La5 × 10^2^ CFU/mL; La5 × 10^4^ means initial inoculum with La5 × 10^4^ CFU/mL; and La5 × 10^6^ means initial inoculum with La5 × 10^6^ CFU/mL.

Figure 2 shows the total counts of all microorganisms included within the biofilm model. La5 × 10^4^ and La5 × 10^6^ significantly reduced the biofilm amount when compared to biofilm without any treatment (*p* ≤ 0.05). Data from the La5 × 10^2^ were not significant (*p* ≥ 0.05) to any of the other groups; therefore, this group was excluded from the next analysis.

**Figure 3:**
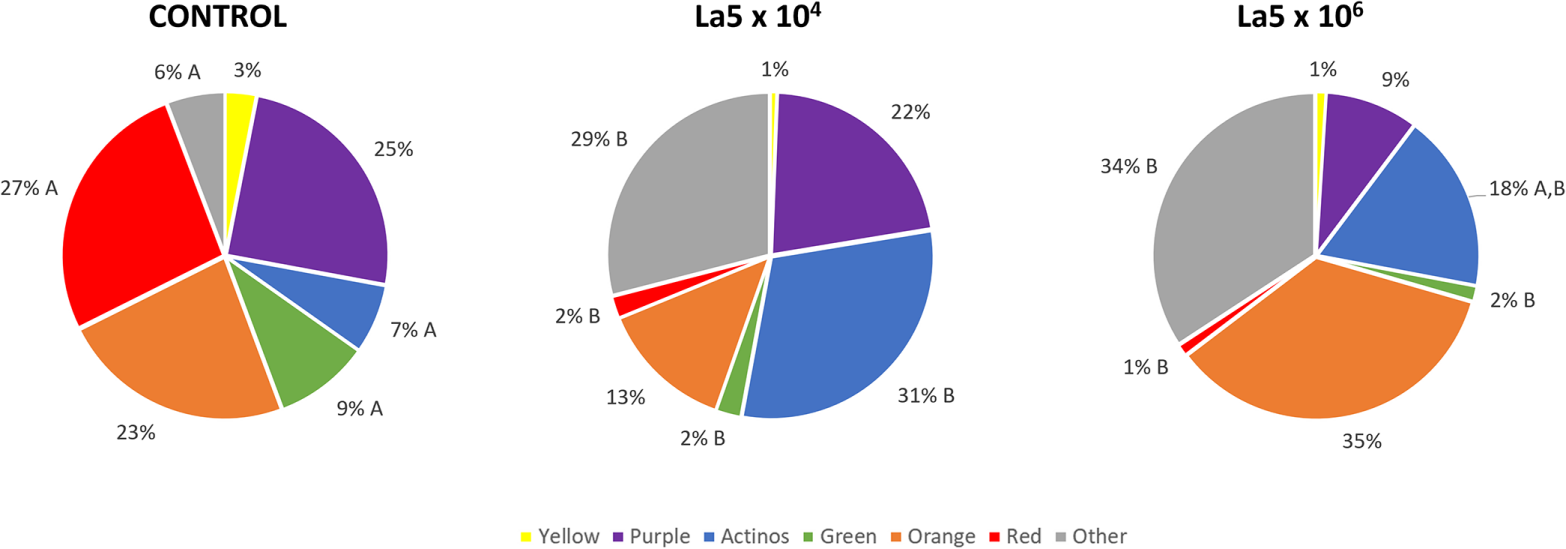
Mean proportions of bacterial complexes multispecies biofilm without any treatment (control) and treated with La5 × 10^4^ and La5 × 10^6^. The colors represent different microbial complexes described by Socransky et al. (17). Different letters mean statistical significance among groups within the same complex. Statistical analysis performed using Kruskal–Wallis followed by Dunn *post hoc* (*p* ≤ 0.05). La5 × 10^2^ means initial inoculum with La5 × 10^2^ CFU/mL; La5 × 10^4^ means initial inoculum with La5 × 10^4^ CFU/mL; and La5 × 10^6^ means initial inoculum with La5 × 10^6^ CFU/mL.

Figure 3 shows the La5 effects on bacterial complexes, as determined by Socransky et al. (19). Both La5 × 10^4^ and La5 × 10^6^ significantly decreased proportions of pathogens in the red complex and those of the beneficial green complex to a very similar number when compared to control (*p* ≤ 0.05). On the other hand, both La5 × 10^4^ and La5 × 10^6^ significantly increased the proportions of other complexes (*p* ≤ 0.05). Finally, only La5 × 10^4^ increased proportions of bacteria in the health-associated actinos complex when compared to the control group (*p* ≤ 0.05).

**Figure 4:**
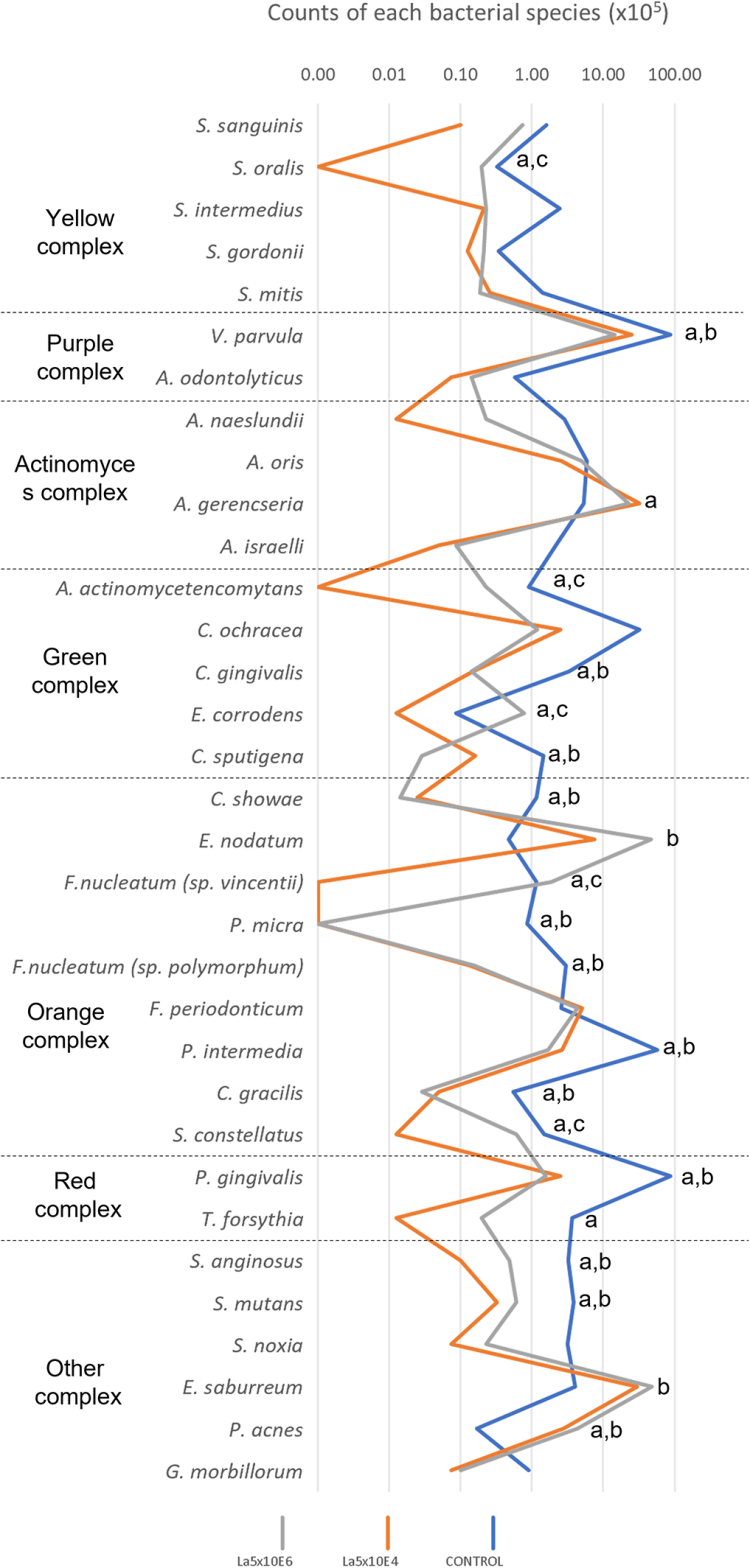
Mean total counts of the species included in the biofilm without any treatment (control) and treated with La5 × 10^4^ and La5 × 10^6^. Statistical analysis was performed by the Kruskal–Wallis test followed by Dunn *post hoc* test (*p* ≤ 0.05). Letter “a” indicates statistical difference between La5 × 10^4^ and control but without statistical difference between La5 × 10^4^ and La5 × 10^6^; “b” indicates statistical difference between La5 × 10^6^ and control but without statistical difference between La5 × 10^4^ and La5 × 10^6^; and “c” indicate statistical difference among La5 × 10^4^ and the two other groups.

Figure 4 demonstrates the results of the counts of each bacterial species within multispecies biofilm. La5 × 10^4^ significantly diminished the counts of 20 species while La5 × 10^6^ decreased the counts of 14 species (*p* ≤ 0.05) when compared to counts of bacterial species within biofilm without any treatment. Both treatment groups share inhibitory effects on 12 species, highlighting the effects on *P. gingivalis* (red complex), *Campylobacter showae*, *Campylobacter gracilis*, *Parvimonas micra*, *Fusobacterium nucleatum polymorphum*, and *Prevotella intermedia* (members of the orange complex). Of even greater impact, only La5 × 10^4^ significantly reduced the counts of *T. forsythia*, another member of the red complex, and *Fusobacterium periodonticum*, a member of the orange complex. In contrast, La5 × 10^6^ increased the counts of *Eubacterium nodatum*, a member of the orange complex. When comparing both La5 treatments, La5 × 10^4^ significantly reduced the counts of *Streptococcus oralis*, *Aggregatibacter actinomycetencomytans*, *Eikenella corrodens*, *Fusobacterium nucleatum vicentii*, and *Streptococcus constellatus* (*p* ≤ 0.05). Therefore, the subgingival multispecies biofilm formed in the presence of La5 × 10^4^ presented lower counts of three major periodontic pathogens such as *P. gingivalis*, *T. forsythia*, and *A. actinomycetencomytans*.

## 4. Discussion

Periodontitis is a chronic multifactorial inflammatory disease associated with a mainly structured biofilm, composed of specific microorganisms and their products, that may guide to tissue damage (20). Herein, *L. acidophilus* reduced biofilm total counts, the red complex proportion, and the amount of the mainly periodontopathogens, such as *P. gingivalis*, *T. forsythia*, *P. micra*, *F. nucleatum polymorphum*, *P. intermedia*, and *A. actinomycetencomytans*, within a subgingival multispecies biofilm model.

The subgingival biofilm is considered the main etiological factor of periodontal disease. The classical work by Socransky et al. (17) grouped bacterial species in the subgingival biofilm into microbial complexes. The yellow (*Streptococcus* spp.), green (*Campylobacter* spp.), purple (*V. parvula* and *A. odontolyticus*), and actinos (*Actinomyces* spp.) complexes were associated with periodontal healthy conditions, while the orange complex (*P. micra*, *Fusobacterium* spp., and *P. intermedia*) was associated to transition from health to disease. Finally, the red complex (*P. gingivalis*, *T. forsythia*, and *Treponema denticola*) was associated with diseased conditions of the periodontum. Although nowadays it is known that the presence of bacterial species in periodontal-diseased sites is much more diverse than the 40 species included in the Socransky complexes (21), this analysis is still an excellent parameter to evaluate antimicrobial effects until new knowledge establish novel periodontal pathogens.

Currently, an agent acting only on the pathogens and their virulence factors is preferable to a broad-spectrum antimicrobial agent since some bacterial species are associated with healthy conditions (22). In this way, both La5 × 10^4^ and La5 × 10^6^ reduced the red complex from 27% to 2% and 1%, respectively. This is a considerable reduction in the same levels observed with the aid of well-known antimicrobials, such as chlorhexidine and cetylpyridinium chloride (23). In line with the current concept, La5 × 10^4^ increased proportions of actinomyces complex associated with healthy conditions.

The present data corroborate the literature that shows that *L. acidophilus* diminishes the *P. gingivalis* abundance within mono and three-species biofilm (24). In addition, the quantities of an *A. actinomycetencomytans* monospecies biofilm were reduced by *L. acidophilus* La5 (13). Thus, *L. acidophilus* La5 has a potential effect as an antibiofilm agent, increasing the scientific basis for future clinical studies for the treatment of periodontitis.

*P. gingivalis*, *T. forsythia*, and possibly other oral bacteria species have been recently indicated as strategic actors in the dysbiosis of the subgingival biofilm, leading to periodontal disease. The presence of these microorganisms can stimulate the transition from a health-associated biofilm to a pathogenic one and start the destruction of tissue due to an increased immunoinflammatory reaction (25). In these circumstances, the reduction of both bacteria by La5 × 10^4^ is an outstanding result, explained by the physical proximity of microorganisms within the biofilm that increases the probability of synergistic or antagonistic interactions.

*Lactobacillus* ssp. produces several antimicrobial compounds, such as hydrogen peroxide, lactate, teichoic acid, and bacteriocins (26, 27), that can inhibit a range of microorganisms, such as *P. gingivalis* and *A. actinomycetencomitans*. Moreover, studies show that lactobacilli can alter the transcription profile of *P. gingivalis* and *A. actinomycetencomitans*, thus interfering in their ability to colonize the host tissues and subvert the immune response; for example, by downregulating the expression of *fimA*, an important virulence factor-related fimbriae formation of *P. gingivalis*, and reduction of leukotoxin (*ltxA*) produced by *A. actinomycetencomitans*.

Another important finding is the reduction of all species of Fusobacterium genera present in the model (*F. nucleatum vincentii*, *F. nucleatum polymorphum*, and *F. periodonticum*) by La5 × 10^4^. The genera *Fusobacterium* plays a relevant role in the transition from periodontal health to disease (28). *Fusobacterium nucleatum* is indicated as the most prevalent anaerobic, Gram-negative species in the late periods of the disease and has been considered a possible periodontal pathogen (29). Some authors (29, 30) have reported that the presence of *F. nucleatum* is mainly associated with individuals with periodontitis and periodontal abscesses, and its levels are reduced after effective periodontal therapy. As an intermediate colonizer of dental biofilm and one of the first Gram-negative species to be stable in the subgingival biofilm, *Fusobacterium* species play an important role in the interactions between Gram-positive and Gram-negative species, contributing to the colonization of other anaerobic species, including the pathogens of the red complex (28).

The limitations of this study include the absence of *Treponema denticola* in the model. Although relevant to the development of periodontitis, previous articles using the same model did not include it due to the difficulty of growing this bacterium *in vitro*. Another limitation is the time of contact of LA5 with the biofilm. How to administer the lactobacilli *in vivo* to keep it stuck to the biofilm from the beginning of periodontal multispecies biofilm development? These are challenges to be overcome in future studies.

The inflammatory response plays a crucial role in the tissue destruction occurring during periodontal disease and probiotics, in addition to exerting action in the colonization of pathogens, can modulate the exacerbated immune host response (31). *In vitro* studies using GECs (11, 12) and human macrophages (32) showed the downregulation of inflammatory cytokines when cells were challenged either with *P. gingivalis* or *A. actinomycetencomitans* and treated with lactobacilli, such as interleukin-1β, a cytokine involved in bone resorption under pathological conditions. Other cytokines/chemokines also presented reduced levels by La5 such as CXCL-8, GM-CSF, and TNF-α. This immunomodulatory response accompanied by the antibiofilm effect indicate that this probiotic strain is a potential candidate for adjunctive therapy in periodontal treatment.

Furthermore, a recent meta-analysis showed that using probiotics as an adjunctive therapy promoted a clinical attachment level gain and reduction of probing pocket depth at 3 and 12 months, which are the main clinical goals in periodontal treatment (33). In addition, a systematic review concluded that administering probiotics as an adjuvant treatment improved the clinical parameters and decreased the concentration of the main periodontal pathogens without causing any side effects (33).

To limit the use of antibiotics and the risk of bacterial resistance, as well as to avoid undesirable effects by repeated therapy, efforts to optimize therapeutic procedures addressing the microbial colonization and recolonization of the periodontal pocket are crucial. Probiotics seem to be a reasonable alternative and our study elucidates that the co-culture of *L. acidophilus* La5 in a multispecies biofilm is capable of reducing the red complex and increasing the *Actinomyces* complex, providing an exciting strategy for the control of dysbiosis. However, more studies elucidating their mechanism of action and the correct timing, quantity, and which strain to be used are still necessary.

## Data Availability

The raw data supporting the conclusions of this article will be made available by the authors, without undue reservation.
